# Do worsening lung ultrasound scans identify severe COVID-19 trajectories?

**DOI:** 10.3389/fmed.2022.1021929

**Published:** 2022-11-21

**Authors:** Paul W. Blair, Jimin Hwang, Jackson Pearce, Tiffany C. Fong, Erjia Cui, Phabiola Herrera, Gigi Liu, Ciprian Crainiceanu, Trishul Siddharthan, Danielle V. Clark, The CCPSEI Research Team, Katherine Fenstermacher

**Affiliations:** Department of Emergency Medicine, Johns Hopkins University, Baltimore, MD, United States; Varun Mahadevan and Joshua East, Division of Pulmonary and Critical Care Medicine, Johns Hopkins University School of Medicine, Baltimore, MD, United States.; ^1^Henry M. Jackson Foundation for the Advancement of Military Medicine, Inc., Bethesda, MD, United States; ^2^Division of Infectious Diseases, The Johns Hopkins University School of Medicine, Baltimore, MD, United States; ^3^Department of Medicine, University of Texas Southwestern Medical Center, Dallas, TX, United States; ^4^College of Medicine, Medical University of South Carolina, Charleston, SC, United States; ^5^Department of Emergency Medicine, Johns Hopkins University, Baltimore, MD, United States; ^6^Department of Biostatistics, Johns Hopkins Bloomberg School of Public Health, Baltimore, MD, United States; ^7^Division of Pulmonary and Critical Care Medicine, University of Miami, Miami, FL, United States; ^8^Division of General Internal Medicine, The Johns Hopkins University School of Medicine, Baltimore, MD, United States; ^9^Division of Pulmonary and Critical Care Medicine, The Johns Hopkins University School of Medicine, Baltimore, MD, United States

**Keywords:** lung ultrasound, point-of-care lung ultrasound, COVID-19, severe COVID-19, cohort study

## Abstract

**Background:**

While point-of-care ultrasound (POCUS) has been used to track worsening COVID-19 disease it is unclear if there are dynamic differences between severity trajectories.

**Methods:**

We studied 12-lung zone protocol scans from 244 participants [with repeat scans obtained in 3 days (*N* = 114), 7 days (*N* = 53), and weekly (*N* = 9)] ≥ 18 years of age hospitalized for COVID-19 pneumonia. Differences in mean lung ultrasound (LUS) scores and percent of lung fields with A-lines over time were compared between peak severity levels (as defined by the WHO clinical progression scale) using linear mixed-effects models.

**Results:**

Mean LUS scores were elevated by 0.19 (*p* = 0.035) and A-lines were present in 14.7% fewer lung fields (*p* = 0.02) among those with ICU-level or fatal peak illness compared to less severe hospitalized illness, regardless of duration of illness. There were no differences between severity groups in the trajectories of mean LUS score 0.19 (*p* = 0.66) or percent A-lines (*p* = 0.40).

**Discussion:**

Our results do not support the use of serial LUS scans to monitor COVID-19 disease progression among hospitalized adults.

## Background

Serial point-of-care lung ultrasound (LUS) provides actionable results at the point-of-care without ionizing radiation. LUS has been an essential tool in evaluating patients with COVID-19 pneumonia, albeit with heterogenous uptake based on center expertise. In contrast to serial chest X-rays, which are no longer standard of care, serial LUS scans are performed without radiation exposure, are more sensitive for detecting lung pathology, and therefore may be more useful as a daily measurement.

While multiple studies (1–5) have assessed the prognostic value of LUS, few (6, 7) have assessed changes over time in a methodical manner. In addition to identifying resolving of severe pulmonary disease, changes in LUS findings could help monitor patient trajectories. However, the variability or trends over time have not been well-described. In the present study we evaluate the association between LUS characteristics (i.e., A-lines, B-lines, consolidations, pleural effusions, pleural line thickening, and a composite score averaged across lung zones) clinical severity among adults hospitalized with COVID-19.

## Methods

**FIGURE 1:**
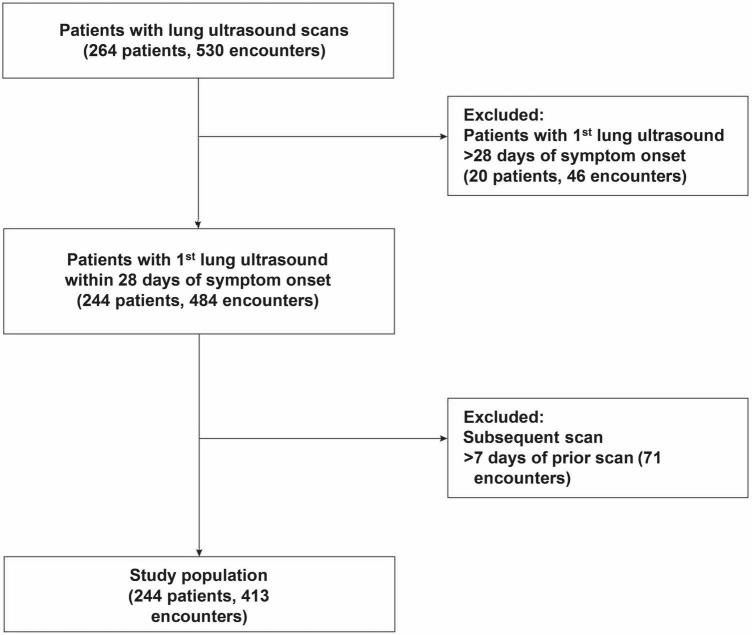
Flow diagram of study population.

We conducted a prospective enrollment of adults age ≥ 18 who were admitted to Johns Hopkins University Hospital and tested positive for SARS-CoV-2, in a larger COVID-19 prospective cohort (ClinicalTrials.gov number, NCT04496466), from April 2020 to September 2021. This protocol was approved by the Johns Hopkins University Institutional Review Board (IRB00245545). After screening SARS-CoV-2 RT-PCR positive patients, a convenience sample of 264 patients were enrolled depending on LUS-trained research staff availability as previously described (1). After enrollment, study visits including LUS scans occurred until hospital discharge on study days 3, 7, and weekly for up to 90 days from enrollment. To evaluate the value of serial scans, our analysis was restricted to 244 participants (413 scanning encounters) after excluding those with an initial scan at >28 days of symptom onset, only one scan, or those with subsequent scans > 7 days of the preceding scan (Figure 1).

As described (1), lung images were collected using 6-s clips from 12 zones using a Lumify S4 phase array probe (Philips, Amsterdam, Netherlands). Study personnel were subsequently masked to clinical information and provided reads identifying and characterizing A-lines (an indicator of normal lung), B-lines (an indicator of edema, fibrosis, or inflammation), consolidations, pleural effusions, and pleural line thickening. Due to a high number of incomplete studies given the hospitalized and intubated status of many patients, a minority (*N* = 58; 23.8%) had at least one complete LUS scan with all 12 zones. Therefore, the mean LUS (mLUS) score was calculated, including those with less than a full 12-zone exam. This composite score (ranging from 0 to 3 with a higher score signifying higher severity) is an average across zones with 1 point for discrete B-lines, 2 points for coalescent B-lines, and 3 points for lung consolidation as previously described (1). Mean number of lung zones scanned across all participants and visits was 5.6.

Participants were grouped by the highest severity reached at the peak of their illness (i.e., moderate or severe) with severe disease defined by requiring high flow nasal cannula, mechanical ventilation, or fatal cases based on the WHO clinical progression scale (8). Disease worsening was considered moving from a moderate level to a severe level and resolving disease was consider moving from a severe level to a moderate level of disease. Summary statistics were calculated for each week post-symptom onset. For individuals with multiple time points during a given week post-symptom onset, a time point was selected at random for each week post-symptom onset to decrease risk of confounding for summary statistics. Differences in mLUS score or percent A-lines changes over time (days post-symptom onset) between peak severity groups were evaluated using linear mixed-effects models. As a sensitivity analysis, models were also run using generalized estimating equations (GEE) and using severity as an ordinal covariate (moderate or severe). Data were analyzed using statistical analytical software Stata version 15.0 (StataCorp LLC, College Station, TX, USA) and figures were created in R (v3.6.3) using the “ggplot” (v3.3.3) package (v1.0.7).

## Results

The cohort included 244 total participants [mean age of 58.2 (SD 15.0) years, and 55.7% female] with 199 participants at 0–14 days post-symptom onset at baseline scan and 45 participants at 15–28 days post-symptom onset. The median LUS time was 9.1 days (IQR: 5.0, 12.7 days) from symptom onset. The median time to peak illness was 11.0 days (IQR: 6.2, 18.3 days) from symptom onset among those that developed severe disease.

The distributions of lung zones with A-lines (normal), B-lines (abnormal), and pleural line abnormalities increased by each level peak severity at baseline (Table 1). At the baseline scan, a widely variable percentage of lung fields contained B-lines (median 58.3%, IQR: 33.3, 0.0%) in patients with moderate COVID-19 but a high degree of B-line changes was consistently present among those with severe peak severity (median 83.3%, IQR: 66.7, 100.0%) (Supplementary Table 1). Conversely, participants in the severe group had a lower percent of A-lines (median 63.6%, IQR: 33.3, 85.7%) compared to the moderate group (median 85.7%, IQR: 66.7, 100.0%). The mLUS score median was 1.0 (IQR: 0.5, 1.3) for the overall cohort.

**TABLE 1:** Lung ultrasound findings by week of illness among adult participants hospitalized with COVID-19 stratified by peak severity.

Variables–median (IQR)	Week of illness			
	0–7 days (*N* = 92)	8–14 days (*N* = 140)	15–21 days (*N* = 55)	22–29 days (*N* = 25)
Moderate disease–no.	74	75	33	12
mLUSS	0.750 (0.250, 1.125)	0.875 (0.333, 1.250)	0.667 (0.500, 1.000)	1.000 (0.000, 1.310)
A lines,%	90.9 (75.0, 100.0)	90.9 (66.7, 100.0)	100.0 (60.0, 100.0)	82.9 (63.3, 100.0)
Any B lines,%	46.4 (25.0, 80.0)	62.5 (33.3, 83.3)	58.3 (33.3, 87.5)	72.2 (0.0, 94.4)
Confluent B lines,%	0.0 (0.0, 20.0)	8.3 (0.0, 25.0)	0.0 (0.0, 0.0)	0.0 (0.0, 18.3)
Consolidations,%	0.0 (0.0, 0.0)	0.0 (0.0, 0.0)	0.0 (0.0, 0.0)	0.0 (0.0, 7.1)
Pleural line abnormalities,%	0.0 (0.0, 8.3)	0.0 (0.0, 12.5)	0.0 (0.0, 0.0)	0.0 (0.0, 10.6)
Pleural effusions,%	0.0 (0.0, 8.3)	0.0 (0.0, 0.0)	0.0 (0.0, 0.0)	0.0 (0.0, 0.0)
Severe disease–no.	18	55	37	13
mLUSS	1.000 (0.667, 1.500)	1.000 (0.667, 1.500)	1.250 (1.000, 1.600)	1.400 (1.000, 1.667)
A lines, %	69.0 (42.9, 100.0)	66.7 (50.0, 100.0)	66.7 (16.7, 83.3)	37.5 (25.0, 75.0)
Any B lines, %	73.2 (50.0, 100.0)	75.0 (50.0, 100.0)	100.0 (62.5, 100.0)	100.0 (750, 100.0)
Confluent B lines, %	0.0 (0.0, 25.0)	0.0 (0.0, 33.3)	0.0 (0.0, 40.0)	28.6 (0.0, 50.0)
Consolidations, %	0.0 (0.0, 25.0)	0.0 (0.0, 22.2)	0.0 (0.0, 25.0)	0.0 (0.0, 25.0)
Pleural line abnormalities, %	0.0 (0.0, 14.3)	0.0 (0.0, 16.7)	0.0 (0.0, 37.5)	60.0 (0.0, 75.0)
Pleural effusions, %	0.0 (0.0, 12.5)	0.0 (0.0, 0.0)	0.0 (0.0, 0.0)	25.0 (0.0, 50.0)

mLUSS, mean lung ultrasound score; IQR, interquartile range.

**FIGURE 2:**
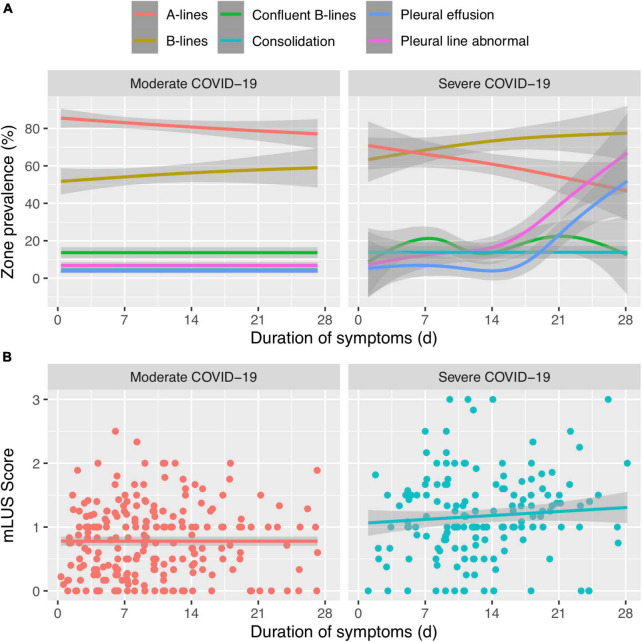
**(A)** Trends of lung ultrasound lung zone involvement (%) over time of different ultrasound artifacts or abnormalities between moderate and severe COVID-19. **(B)** The composite mean LUS (mLUS) score over time between moderate and severe COVID-19. Line is fitted with generalized additive model and the gray line represents standard error.

Stratifying by these peak severity groups, the mLUS score remained higher in severe disease than in moderate disease (Figure 2 and Supplementary Table 1). Regardless of week of illness, more pervasive B-lines were higher in the severe group than in moderate illness and A-lines were lower in severe illness (Supplementary Table 1). Severe COVID-19 patients had consistently higher percent lung zones with B-lines throughout the first month of illness than moderate COVID-19 (Figure 2 and Table 1). Most lung zones were unaffected by pleural line abnormalities in either severity group. A-lines became less prevalent over time among those with moderate or severe disease (Figure 2 and Supplementary Table 1), and B-lines became more prevalent over time among participants that remained hospitalized during their third and fourth week of illness. Consolidations, pleural line abnormalities, and pleural effusions were uncommon throughout illness in both moderate and severe groups (Supplementary Table 1). The percent of lung zones with pleural line abnormalities or pleural effusions was higher in severe disease than moderate at 22–29 days of illness, but sample size was limited to 13 and 12, respectively (Figure 2 and Table 1).

In the linear mixed-effects model, mLUS scores were elevated by 0.19 (*p* = 0.035) in the severe group compared to the moderate group regardless of duration of illness. However, there was no difference in mLUS trajectory (i.e., change over time) between severity groups (*p* = 0.66). A sensitivity analysis using study visits instead of duration of symptoms and another analysis using GEE resulted in the same qualitative conclusion (data not shown). Similarly, A-lines were in 14.7% fewer lung fields (*p* = 0.02; Figure 2) and B-lines were in 17.8% more lung fields among those with severe disease compared to moderate disease regardless of duration of illness. However, the trajectory of percent A-lines did not differ significantly between peak severity levels (*p* = 0.40) and the trajectory of percent B-lines did not differ (*p* = 0.83).

## Discussion

Our study found no significant differences in LUS findings over time between those with severe (i.e., ICU-level or fatal cases) and moderate (non-ICU) peak illness among adults hospitalized with COVID-19. Lung ultrasound abnormalities became increasingly prevalent among the minority of those that remained hospitalized during the third or fourth week of illness. However, the slope of that increase did not differ between moderate or severe disease. While the benefits of portability and bedside results of LUS are appealing, our results did not reveal differences in the composite mLUS scores or percent of zones with A-lines over the patients’ clinical course. Baseline LUS or LUS after a prolonged stay may be more informative than dynamic LUS changes among those hospitalized with COVID-19 pneumonia.

This represents, to our knowledge, the largest study with serial COVID-19 LUS. Prior research has identified similar findings of persistent abnormalities (7) but has not evaluated for changes as potential indicators of a severe disease trajectory. The dearth of differences may be related to persistent architectural changes that may lag clinical improvement similar to that which may occur with chest X-rays or computed tomography (CT) scans in which residual disease observed on medical imaging does not reflect the relative recovery in clinical condition.

There are limitations to our study. Participants were initially scanned after admission to the hospital and earlier changes in lung ultrasound findings may not have been observed. Mean LUS score was used to mitigate the effect of incomplete lung scans of <12 zones. While this may introduce bias, it reflects the real-world application of ultrasound in the clinical care of patients with moderate and severe illness. The small sample size within the third and fourth weeks of illness due to attrition from discharges could have resulted in an inability to detect more subtle differences and included those that were more ill. As participants were not scanned after discharge, our findings during late illness are likely representative of the course for individuals that remain hospitalized for illness due to COVID-19 rather than individuals that are hospitalized for COVID-19 in general. Scans were collected in the pre-omicron era; however, imaging is expected to be similar among those hospitalized with severe COVID-19 due to omicron variants. Differences or sensitivity analyses based on ventilator settings (e.g., positive end-expiratory pressure) were not assessed due to data availability. Further studies are needed in the ambulatory setting to improve understanding of diagnostic accuracy among non-hospitalized individuals with COVID-19.

Mean LUS scores correlated with clinical severity among hospitalized adults when assessed cross-sectionally; however, mLUS score did not change or differ between peak severity levels over the time course of hospitalization. These results do not support serial LUS scans to monitor progression of disease severity. While future studies may identify other potential applications for serial LUS, LUS remains an important tool for clinical care in detecting and diagnosing various lung pathologies.

## Data availability statement

The raw data supporting the conclusions of this article will be made available upon reasonable request to the corresponding author.

## Ethics statement

The studies involving human participants were reviewed and approved by Johns Hopkins University Institutional Review Board (IRB00245545). Written informed consent for participation was not required for this study in accordance with the national legislation and the institutional requirements.

## The Clinical Characterization Protocol for Severe Infectious Diseases (CCPSEI) research team members

Katherine Fenstermacher, Sophia Shea, Stefanie Seo, Josh Lawrence, Lauren Sauer, Bhakti Hansoti, and Richard Rothman, Department of Emergency Medicine, Johns Hopkins University, Baltimore, MD, United States; Varun Mahadevan and Joshua East, Division of Pulmonary and Critical Care Medicine, Johns Hopkins University School of Medicine, Baltimore, MD, United States.

## Author contributions

PB wrote the manuscript and performed the statistical analysis. JH and JP performed the statistical analysis and edited the manuscript. TF edited the manuscript and was involved in concept development. EC and CC provided the statistical support and oversight. PH provided the operational support for data acquisition. GL was involved in data acquisition. TS was part of concept development and data acquisition. DC contributed to the support and concept development. All authors contributed to the article and approved the submitted version.

## Author disclaimer

The contents of this article are sole responsibility of the authors and do not necessarily reflect the views, assertions, opinions, or policies of the Henry M. Jackson Foundation for the Advancement of Military Medicine, Inc., the U.S. Department of Defense, the U.S. government, or any other government or agency. Mention of trade names, commercial products, or organizations does not imply endorsement by the U.S. government. Some of the authors of this work are military service members or employees of the U.S. government. This work was prepared as part of their official duties. Title 17 U.S.C. x105 provides that “Copyright protection under this title is not available for any work of the United States government”. Title 17 U.S.C. x101 defines a U.S. government work as a work prepared by a military service member or employee of the U.S. government as part of that person’s official duties. The investigators have adhered to the policies for protection of human subjects as prescribed in 45 CFR 46.
