# Supplementary material for: Do worsening lung ultrasound scans identify severe COVID-19 trajectories?
Source: Front Med (Lausanne). 2022 Nov 21;9:1021929. doi: 10.3389/fmed.2022.1021929 (PMC9720255; doi:10.3389/fmed.2022.1021929)
Supplement: Supplementary file 1 [file Table_1.pdf]

Table S1. Baseline demographic and lung ultrasound characteristics of the population.

**Variables–median (IQR)**	**Overall (N=244)**	**Moderate at baseline (N=165)**	**Severe at baseline (N=79)**
Age–median (IQR)	60.5 (48.0, 68.0)	60.0 (44.0, 68.0)	62.0 (53.0, 68.0)
Female–no. (%)	108 (44.3)	83 (50.3)	25 (31.7)
Race–no. (%)			
Black	120 (49.2)	91 (55.2)	29 (36.7)
White	73 (29.9)	46 (27.9)	27 (34.2)
Asian	7 (2.9)	1 (0.6)	6 (7.6)
American/Alaska Native	2 (0.8)	2 (1.2)	0 (0)
Other	40 (16.4)	24 (14.6)	16 (20.3)
Not available	2 (0.8)	1 (0.6)	1 (1.3)
Hispanic Race	37 (15.2)	24 (14.6)	13 (16.5)
BMI–median (IQR)*	29.0 (25.4, 33.2)	27.5 (24.0, 32.9)	30.5 (26.9, 35.1)
Symptom onset to LUS time (days)–median (IQR)	9.1 (5.0, 12.7)	7.4 (4.1, 11.8)	10.3 (8.2, 15.2)
Ultrasound parameters			
Mean LUS score – median (IQR)	1.0 (0.5, 1.3)	0.8 (0.4, 1.2)	1.0 (0.9, 1.6)
A line proportion –%, median (IQR)	75.0 (55.8, 100.0)	85.7 (66.7, 100.0)	63.6 (33.3, 85.7)
Any B line proportion –%, median (IQR)	66.7 (36.9, 95.8)	58.3 (33.3, 80.0)	83.3 (66.7, 100.0)
Confluent B line proportion–%, median (IQR)	4.2 (0.0, 27.9)	0.0 (0.0, 25.0)	8.3 (0.0, 33.3)
Consolidation proportion–%, median (IQR)	0.0 (0.0, 8.3)	0.0 (0.0, 8.3)	0.0 (0.0, 16.7)
Pleural effusion–%, median (IQR)	0.0 (0.0, 8.3)	0.0 (0.0, 8.3)	0.0 (0.0, 0.0)
Pleural line abnormality–%, median (IQR)	0.0 (0.0, 16.7)	0.0 (0.0, 12.5)	0.0 (0.0, 33.3)

*BMI was missing from 41 participants.
